# Endothelin-1 Induces Cell Proliferation and Myofibroblast Differentiation through the ET_A_R/G_αq_/ERK Signaling Pathway in Human Cardiac Fibroblasts

**DOI:** 10.3390/ijms24054475

**Published:** 2023-02-24

**Authors:** Ratchanee Duangrat, Warisara Parichatikanond, Sutharinee Likitnukul, Supachoke Mangmool

**Affiliations:** 1 Department of Pharmacology, Faculty of Science, Mahidol University, Bangkok 10400, Thailand; 2 Molecular Medicine Graduate Program, Faculty of Science, Mahidol University, Bangkok 10400, Thailand; 3 Department of Pharmacology, Faculty of Pharmacy, Mahidol University, Bangkok 10400, Thailand; 4 Centre of Biopharmaceutical Science for Healthy Ageing (BSHA), Faculty of Pharmacy, Mahidol University, Bangkok 10400, Thailand

**Keywords:** α-SMA, ambrisentan, bosentan, cardiac fibrosis, collagen, endothelin-1, ET_A_R, human cardiac fibroblast, myofibroblast differentiation

## Abstract

Endothelin-1 (ET-1) has been implicated in the pathogenesis of cardiac fibrosis. Stimulation of endothelin receptors (ETR) with ET-1 leads to fibroblast activation and myofibroblast differentiation, which is mainly characterized by an overexpression of α-smooth muscle actin (α-SMA) and collagens. Although ET-1 is a potent profibrotic mediator, the signal transductions and subtype specificity of ETR contributing to cell proliferation, as well as α-SMA and collagen I synthesis in human cardiac fibroblasts are not well clarified. This study aimed to evaluate the subtype specificity and signal transduction of ETR on fibroblast activation and myofibroblast differentiation. Treatment with ET-1 induced fibroblast proliferation, and synthesis of myofibroblast markers, α-SMA, and collagen I through the ET_A_R subtype. Inhibition of G_αq_ protein, not G_αi_ or G_βγ_, inhibited these effects of ET-1, indicating the essential role of G_αq_ protein-mediated ET_A_R signaling. In addition, ERK1/2 was required for ET_A_R/G_αq_ axis-induced proliferative capacity and overexpression of these myofibroblast markers. Antagonism of ETR with ETR antagonists (ERAs), ambrisentan and bosentan, inhibited ET-1-induced cell proliferation and synthesis of α-SMA and collagen I. Furthermore, ambrisentan and bosentan promoted the reversal of myofibroblasts after day 3 of treatment, with loss of proliferative ability and a reduction in α-SMA synthesis, confirming the restorative effects of ERAs. This novel work reports on the ET_A_R/G_αq_/ERK signaling pathway for ET-1 actions and blockade of ETR signaling with ERAs, representing a promising therapeutic strategy for prevention and restoration of ET-1-induced cardiac fibrosis.

## 1. Introduction

Cardiac fibrosis contributes to the abnormality of cardiac functions, leading to the development of cardiac remodeling and progression of heart failure (HF) [1,2]. The mortality and morbidity rates remain high despite recent advances in the HF therapy. HF is still a major global health problem, with the worldwide prevalence of over 64.3 million people in 2017 [3]. After cardiac injury, cardiac fibroblasts can be induced by many profibrotic factors including endothelin-1 (ET-1) in the activated form, resulting in increased fibroblast proliferation, extracellular matrix (ECM) proteins deposition, and myofibroblast differentiation [1,4,5]. Myofibroblasts are active cells which characterized by overproduction of α-smooth muscle actin (α-SMA) [5,6]. Fibroblast proliferation and myofibroblast differentiation are the hallmarks of a fibrotic response in the heart.

ET-1 is upregulated and secreted in the heart of HF patients [7] and animal models of cardiac fibrosis [8], indicating that ET-1 is a potent fibrogenic mediator. Stimulation of endothelin receptors (ETRs) with ET-1 induces myocardial fibrosis and is associated with cardiac abnormalities, leading to HF [9]. In rat cardiac fibroblasts, ET-1 induced cell proliferation, deposition of ECM, and α-SMA production as well as promoted the transdifferentiation of fibroblasts into myofibroblasts [10,11]. In contrast, myocardial fibrosis was attenuated in mice with vascular endothelial cell-specific ET-1 deficiency [12].

ETR is one of the G protein-coupled receptor (GPCR) superfamilies, denoted by endothelin receptor type A (ET_A_R) and type B (ET_B_R) [13]. Stimulation of ETRs with ET-1 promotes cell proliferation, adhesion, vasoconstriction, and fibrosis in various types of cells and tissues [13,14]. ET_A_R was involved in the development of fibrosis in deoxycorticosterone acetate (DOCA)-induced hypertensive rats, while ET_B_R mediated protective effects against vascular and renal injuries [15]. Furthermore, ET-1 induced myofibroblast differentiation and collagen matrix contraction through the ET_A_Rs, but not ET_B_Rs, in lung fibroblasts [16]. ET-1-mediated ET_A_R signaling was associated with upregulation of fibronectin and matrix metalloproteinases (MMPs), as well as collagen deposition in the heart of hypertensive rats [17]. However, both ET_A_R and ET_B_R are necessary for the fibrotic effects of ET-1 by contributing to the proliferation of lung fibroblasts [18].

After ET-1 binding, the ETRs can couple to heterotrimeric G_q_ proteins, leading to the dissociation of G_αq_ and G_βγ_ subunits to stimulate their effectors. Consequently, G_αq_ proteins activate phospholipase C (PLC) activity, leading to the synthesis of inositol 1,4,5-trisphosphate (IP_3_) and diacylglycerol (DAG) [19]. IP_3_ then induces the elevation of intracellular Ca^2+^ levels, while DAG causes protein kinase C (PKC) translocation, resulting in activation of Ras proteins, and consequently, the extracellular signal-regulated kinase (ERK) signaling pathway [20]. For example, ET-1 transduced the signaling to activation of the ERK1/2 via G_q/11_ protein-dependent pathway in L6 myoblasts [21]. Interestingly, ETRs can couple with other G_α_ proteins, including G_αi_ proteins [19,22]. In addition to G_α_ proteins, the G_βγ_ subunit of ET_B_R provoked the PI3K/Akt/eNOS signaling pathway, resulting in nitric oxide production in endothelial cells [23]. Thus, ETRs could activate multiple signaling pathways for the induction of cardiac fibrosis, which displayed unique effects depending on receptor subtypes and G_α_ protein isoforms. However, it remains to be elucidated which ETR subtypes and G_α_ protein isoforms are involved in the ET-1-induced fibroblast stimulation and myofibroblast differentiation in human cardiac fibroblasts. In this study, we aimed to investigate how the ETR signaling pathway contributed to an induction of cardiac fibrosis. The identification of the molecular mechanisms by which ETR mediates fibroblast activation and myofibroblast differentiation (or transformation) will help us to understand this underlying pathway. Therefore, the antagonism of ETR and blockade of related signaling have been proposed to be the potential therapeutic strategy for prevention and/or treatment of cardiac fibrosis.

The important downstream effectors of ETRs include mitogen-activated protein kinases (MAPKs), such as c-Jun N-terminal kinases (JNKs), ERKs, and p38 MAPKs that contribute to the pathology of fibrotic diseases [19,22]. In vascular smooth muscle cells (VSMCs), blockade of ERK activity diminished ET-1-induced connective tissue growth factor (CTGF) production, whereas blockade of p38 MAPK had no effect [24]. Treatment with ET-1 induced ERK1/2 activation in rat cardiac myocytes [25], indicating that ERK1/2 is required for myocardial hypertrophy induced by ET-1. Moreover, ET_A_R regulated ERK1/2 activation via the G_q/11_ protein-dependent and PLC-independent pathway in L6 myoblasts, revealing the minor contribution of PLC to ET-1-induced activation of ERK1/2 [21]. The importance of ERK1/2 for ETR signaling has been demonstrated in various types of cells; however, whether ERK1/2 is required for fibrotic effects of ET-1 in human cardiac fibroblasts has not been clearly identified.

Blockade of ETR signaling has been demonstrated to alleviate cardiac fibrosis in DOCA-induced hypertensive rats [26] and in rats with pressure overload-induced hypertrophy [27]. In addition, bosentan (a nonselective ERA) has been shown to attenuate myocardial fibrosis and remodeling in rats with myocardial infarction [28]. Long-term treatment with bosentan increased the survival rate in rats with chronic HF [29]. Administration of bosentan to HF patients led to the improvement of cardiac functions, resulting in hemodynamic benefits [30]. Interestingly, most HF patients reach clinical attention only after significant fibrosis has already been found, so reversibility of the myofibroblast differentiation process might serve as a promising tool to restore the pathology of fibrosis. In the present study, we also investigated the preventive and restorative effects of ambrisentan and bosentan on ET-1-induced fibroblast activation and myofibroblast differentiation.

## 2. Results

### 2.1. ET-1 Induces Cell Proliferation, and Expression of α-SMA and Collagen I in a Dose-Dependent Manner

**Figure 1:**
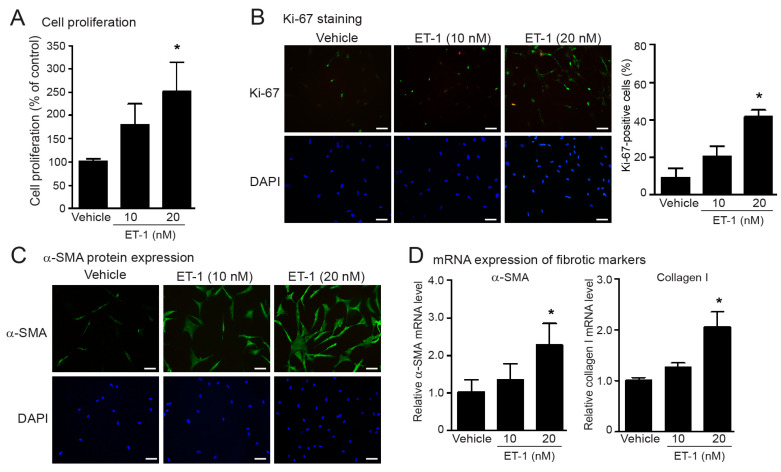
Treatment with ET-1 induced cell proliferation, and α-SMA and collagen I synthesis in human cardiac fibroblasts. (**A**–**D**) Cells were treated with various concentrations of endothelin-1 (ET-1) for 24 h (**A**–**C**) and 48 h (**D**). (**A**) Cell proliferation was calculated as the percentage relative to control group, and expressed as the mean ± SD. (**B**) Proliferative capacity of fibroblasts was determined by Ki-67 immunofluorescence assay. Cells were stained for Ki-67 (green) and nucleus DAPI (blue). Scale bar represents 10 µm. (**C**) α-SMA protein expression was visualized by fluorescent microscope. Cells were stained for α-SMA (green) and nucleus DAPI (blue). Scale bar represents 10 µm. (**D**) Relative α-SMA and collagen I mRNA levels determined by qRT-PCR were calculated as fold over the vehicle-treated group, and expressed as the mean ± SD. Data are obtained from four independent repetitions (*n* = 4). * *p* < 0.05 versus vehicle.

We first assessed the profibrotic effects of ET-1 on fibroblast activation and myofibroblast differentiation as determined by cell proliferation, and collagen I and α-SMA synthesis in fetal human cardiac fibroblasts. Cells were incubated with different doses of ET-1 (1–20 nM) for 24 h. Stimulation of ETRs with ET-1 led to a significant increase in the amounts of fibroblasts in a dose-dependent manner that showed the highest efficacy at a dose of 20 nM (Figure 1A). To further identify the proliferative capacity of ET-1, we used Ki-67 nuclear staining as a marker of proliferation. The density of Ki-67-positive fibroblast cells was generally low in non-treated fibroblasts (Figure 1B). Treatment with ET-1 significantly increased the Ki-67-positive fibroblasts, as shown by the green color in a dose-dependent way (Figure 1B). Myofibroblasts are highly active cells that overexpress α-SMA and collagen I. We next investigated the effects of ET-1 on collagen I and α-SMA synthesis and found that treatment with ET-1 resulted in a significant increase in α-SMA protein and mRNA expression, as well as collagen I mRNA expression in a dose-dependent manner, which showed the maximal effect at the concentration of 20 nM (Figure 1C,D). These results demonstrate that stimulation of ETRs enhanced the cell proliferation and the synthesis of α-SMA and collagen I of human cardiac fibroblasts. In addition, treatment with ET-1 for 24 h and up to 48 h increased the number of fibroblasts as well α-SMA protein expression (Supplementary Figure S1).

### 2.2. ET-1-Induced Fibroblast Proliferation, and Synthesis of α-SMA and Collagen I Mediated through the ET_A_R Subtype

**Figure 2:**
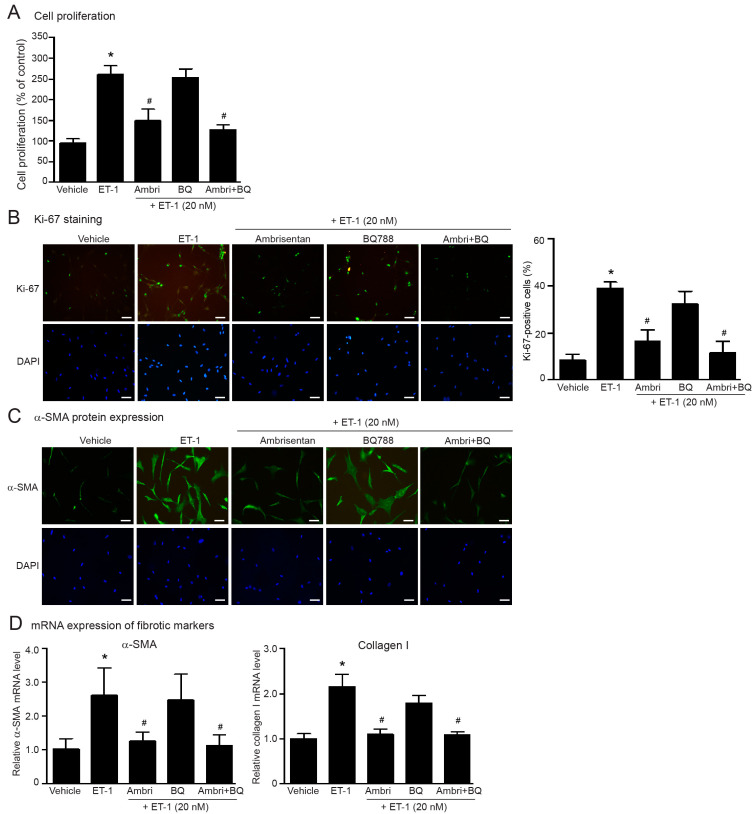
ET-1 induced cell proliferation and overexpression of myofibroblast markers through the ET_A_R subtype. Cells were pretreated without or with ambrisentan (ET_A_R antagonist; 1 µM), BQ-788 (ET_B_R antagonist; 1 µM) or ambrisentan plus BQ-788 for 1 h before treatment with ET-1 for 24 h (**A**–**C**) or 12 h (**D**). (**A**) Cell proliferation was calculated as percentage relative to control group. (**B**) Cells were stained for Ki-67 (green) and nucleus DAPI (blue). Scale bar, 10 µm. Proliferative capacity was calculated as the percentage of Ki-67-positive cells. (**C**) α-SMA protein expression was visualized by fluorescent microscope. Cells were stained for α-SMA (green) and nucleus DAPI (blue). Scale bar, 10 µm. (**D**) Relative α-SMA and collagen I mRNA levels analyzed by qRT-PCR were calculated as the fold over the vehicle-treated group. Data were expressed as the mean ± SD. (*n* = 4). * p < 0.05 vs. vehicle; ^#^ *p* < 0.05 vs. ET-1.

The biological effects of ET-1 are derived from activation of ETRs, which exist in ET_A_Rs and ET_B_Rs. We used a selective ET_A_R antagonist (ambrisentan), and a selective ET_B_R antagonist (BQ-788) to identify which ETR subtypes are associated with the fibrotic effects of ET-1 in human cardiac fibroblasts. Pretreatment with ambrisentan significantly inhibited ET-1-induced cell proliferation (Figure 2A,B), and synthesis of α-SMA and collagen I (Figure 2C,D), whereas BQ-788 had no effects. Blockade of both ETR subtypes completely inhibited these ET-1 effects (Figure 2). Treatment with these antagonists alone had no effect on cell proliferation and did not cause cell cytotoxicity (Supplementary Figure S2). These data revealed that stimulation of ET_A_Rs, not ET_B_Rs, induced fibroblast activation and myofibroblast transdifferentiation.

### 2.3. G_αq_ protein Is Responsible for ET_A_R-Mediated Cell Proliferation and Myofibroblast Differentiation

**Figure 3:**
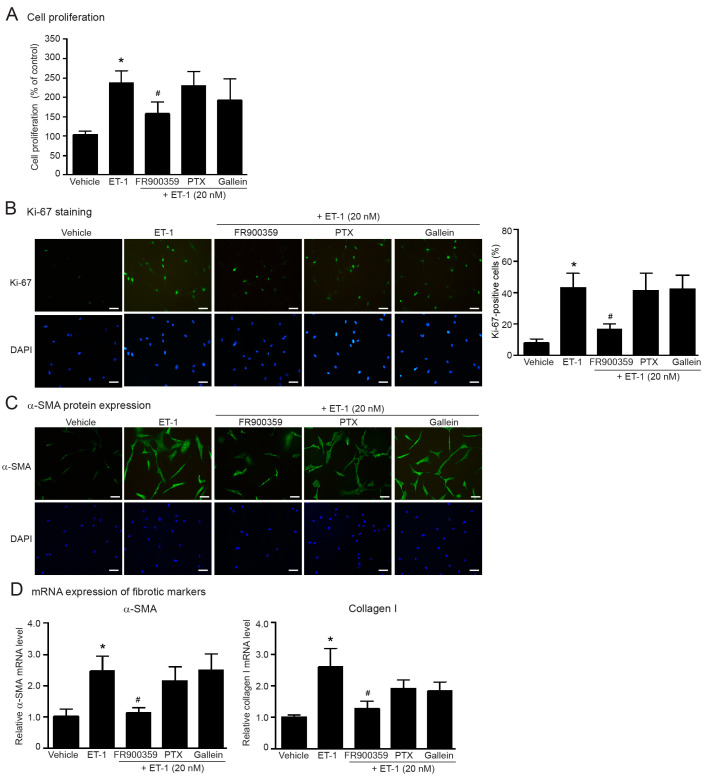
ET-1-induced cell proliferation and synthesis of α-SMA and collagen I is mediated through ET_A_R/G_αq_ signaling. Cells were pretreated without or with FR900359 (G_q_ inhibitor; 5 µM), PTX (G_i/o_ inhibitor; 10 µM) or gallein (G_βγ_ inhibitor; 10 µM) for 1 h before treatment with 20 nM ET-1 for 24 h (**A**–**C**) or 12 h (**D**). (**A**) Cell proliferation was calculated as the percentage relative to control group. (**B**) Cells were stained for Ki-67 (green) and nucleus DAPI (blue). Scale bar, 10 µm. Proliferative capacity was calculated as the percentage of Ki-67-positive cells. (**C**) α-SMA protein expression was visualized by fluorescent microscope. Cells were stained for α-SMA (green) and nucleus DAPI (blue). Scale bar, 10 µm. (**D**) Relative α-SMA and collagen I mRNA levels determined by RT-qPCR were calculated as the fold over the vehicle-treated group. Data were expressed as the mean ± SD. (*n* = 4). * p < 0.05 vs. vehicle; ^#^ *p* < 0.05 vs. ET-1.

ET_A_R is classified as a G_q_ protein-coupled receptor which transduces the signals mediated through heterotrimeric G_q_ proteins. After ET-1 binding, the ET_A_R interact with heterotrimeric G_q_ proteins, leading to the dissociation of the G_αq_ protein from the G_βγ_ subunit [19]. Not only coupling with G_q_ protein, ET_A_Rs also couple with the G_i_ protein [19,22]. Pertussis toxin (PTX) is widely used for inhibition of the G_i/o_ protein by catalyzing the ADP-ribosylation of the α subunit of G_i/o_ protein [31]. We used specific inhibitors of G_α_ protein activities, including FR900359 (G_q_ inhibitor), and PTX (G_i/o_ inhibitor) to identify which isoform of G_α_ proteins mediates ETR signaling. As shown in Figure 3, blockade of G_q_ signaling using FR900359 significantly reduced ET-1-induced fibroblast proliferation and the synthesis of α-SMA and collagen I, whereas inhibition of G_i_ signaling using PTX had no effect.

We also used gallein, a specific G_βγ_ inhibitor, to investigate whether G_βγ_ subunits play a critical role in ET-1-mediated cardiac fibrosis. We found that gallein did not inhibit ET-1 actions (Figure 3). Additionally, these inhibitors had no effect on cell proliferation and did not cause cell cytotoxicity (Supplementary Figure S2). Taken together, these data suggest that ET-1 exhibits profibrotic effects through the ET_A_R/G_αq_ axis.

### 2.4. ERK1/2 Is Necessary for ET-1-Induced Fibroblast Activation and Myofibroblast Differentiation

MAPKs have been associated with ETR signaling. ET-1 can activate MAPKs such as ERKs, JNKs, and p38 MAPKs that contribute to the pathology of fibrotic diseases [19,22]; therefore, we examined whether the profibrotic effects of ET-1 are mediated through MAPKs by using specific inhibitors, SP600125 (JNK inhibitor), FR180204 (ERK1/2 inhibitor), and SB203580 (p38 MAPK inhibitor).

**Figure 4:**
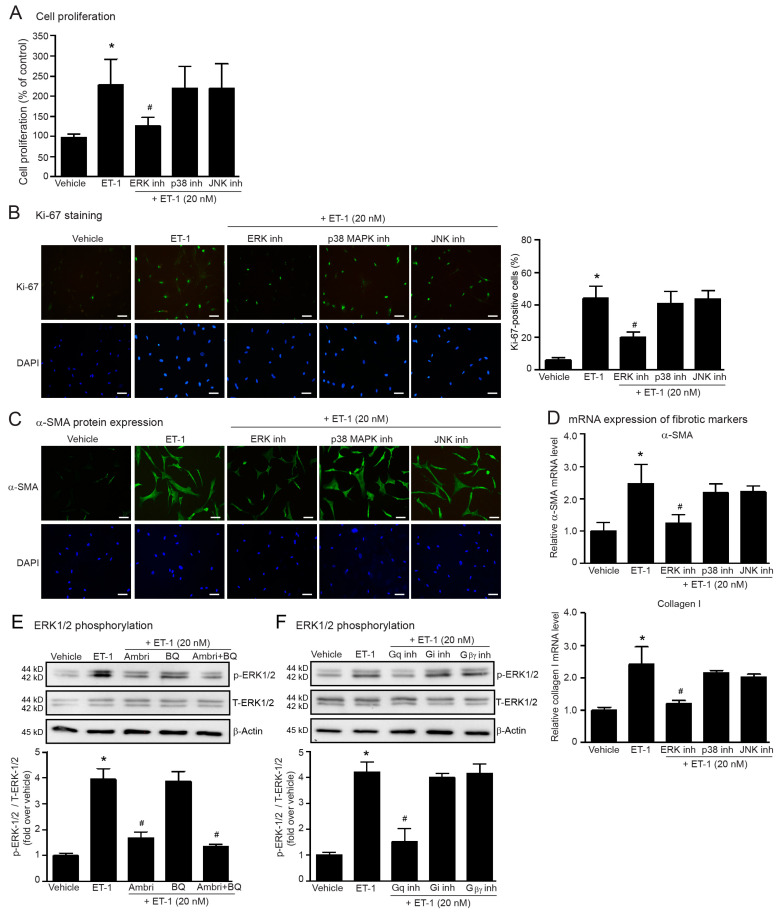
Inhibition of ERK1/2 suppresses ET-1-induced cell proliferation and myofibroblast differentiation. Cells were pretreated without or with 1 µM FR180204 (ERK1/2 inhibitor; ERK inh), 1 µM SB203580 (p38 inhibitor; p38 inh), 1 µM SP600125 (JNK inhibitor; JNK inh) for 1 h before treatment with 20 nM ET-1 for 24 h (**A**–**C**) or 12 h (**D**). (**A**) Cell proliferation was calculated as the percentage relative to the control group. (**B**) Cells were stained for Ki-67 (green) and nucleus DAPI (blue). Scale bar, 10 µm. Proliferative capacity was calculated as the percentage of Ki-67-positive cells. (**C**) α-SMA protein expression was visualized by fluorescent microscope. Cells were stained for α-SMA (green) and nucleus DAPI (blue). Scale bar, 10 µm. (**D**) Relative α-SMA and collagen I mRNA levels determined by RT-qPCR were calculated as the fold over the vehicle-treated group. (**E**) Cells were pretreated without or with ambrisentan (1 µM), BQ-788 (1 µM) or ambrisentan plus BQ-788 for 1 h before treatment with ET-1 for 30 min. (**F**) Cells were pretreated without or with FR900359 (G_q_ inh; 5 µM), PTX (G_i/o_ inh; 10 µM) or gallein (G_βγ_ inh; 10 µM) for 1 h before treatment with ET-1 for 30 min. (**E**,**F**) Cells were pretreated without or with specific inhibitors for 1 h before treatment with 20 nM ET-1 for 30 min. ERK1/2 activation was assessed by the levels of phosphorylated ERK1/2 as compared to total ERK1/2 and calculated as the fold over the vehicle-treated group. Data were shown as the mean ± SD. (*n* = 4). * p < 0.05 vs. vehicle; ^#^ *p* < 0.05 vs. ET-1.

We first tested the cytotoxic effects of each inhibitor and found that SP600125, FR180204, and SB203580 did not cause cell cytotoxicity (Supplemental Figure S2). We found that the blockade of ERK1/2 activity significantly inhibited ET-1-induced fibroblast proliferation (Figure 4A,B), and α-SMA and collagen I synthesis (Figure 4C,D). In contrast, inhibitions of p38 MAPKs and JNKs had no effect on ET-1 actions. Hence, ET-1 stimulation of ETR induced cardiac fibrosis in a ERK1/2-dependent way. Since the treatment with ET-1 could induce ERK1/2 activation in rat cardiac fibroblasts [32,33], we next determined the effects of ET-1 on ERK1/2 activation by measurement of the phosphorylated ERK1/2 levels in human cardiac fibroblasts. Treatment fibroblasts with ET-1 dramatically increased the phosphorylated ERK1/2 (*p*-ERK1/2) levels (Figure 4E). Pretreatment with ambrisentan (a selective ET_A_R antagonist) markedly suppressed the ET-1-induced ERK1/2 phosphorylation, whereas BQ788 (a selective ET_B_R antagonist) had no effect (Figure 4E). Moreover, ET-1-induced ERK1/2 phosphorylation was dramatically reduced by FR900359 (specific G_αq_ inhibitor) (Figure 4F). All original immunoblots were presented in Supplementary Figure S3. However, blockades of G_αi_ signaling using PTX and G_βγ_ signaling using gallein failed to inhibit ET1-induced ERK1/2 phosphorylation (Figure 4F). Taken together, these data suggest that activation of ERK1/2 by ET-1 occurs via an ET_A_R/G_αq_-dependent pathway.

### 2.5. ERAs Prevent ET-1-Induced Fibroblast Proliferation and Myofibroblast Differentiation

**Figure 5:**
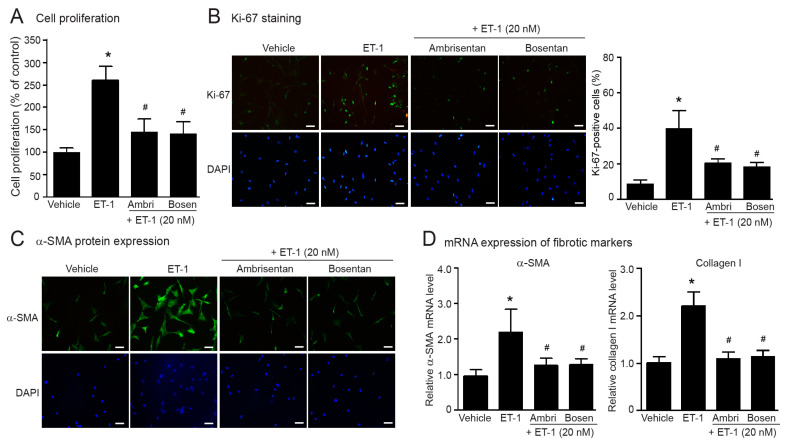
Preventive effects of ERAs on ET-1-induced cell proliferation and myofibroblast differentiation. Cells were pretreated without or with ambrisentan (1 µM) or bosentan (1 µM) for 1 h before treatment with 20 nM ET-1 for 24 h (**A**–**C**) or 12 h (**D**). (**A**) Cell proliferation was calculated as the percentage relative to the control group. (**B**) Cells were stained for Ki-67 (green) and nucleus DAPI (blue). Scale bar, 10 µm. Proliferative capacity was calculated as the percentage of Ki-67-positive cells. (**C**) α-SMA protein expression was visualized by fluorescent microscope. Cells were stained for α-SMA (green) and nucleus DAPI (blue). Scale bar, 10 µm. (**D**) Relative α-SMA and collagen I mRNA levels determined by RT-qPCR were calculated as fold over the vehicle-treated group. Data were expressed as the mean ± SD. (*n* = 4). * p < 0.05 vs. vehicle; ^#^ *p* < 0.05 vs. ET-1.

According to our present data showing the stimulation of ET_A_Rs induced fibroblast activation and myofibroblast differentiation, we next investigated the preventive effects of currently approved ERAs, ambrisentan and bosentan, on the effects of ET-1. Blockade of ET_A_Rs with these ERAs completely inhibited ET-1-induced fibroblast proliferation, and α-SMA and collagen synthesis in human cardiac fibroblasts (Figure 5). Together, these data indicated that ambrisentan and bosentan exhibit preventive effects on the inhibition of ET-1-induced cardiac fibrosis.

### 2.6. ERAs Reverse Myofibroblast Differentiation Induced by ET-1

**Figure 6:**
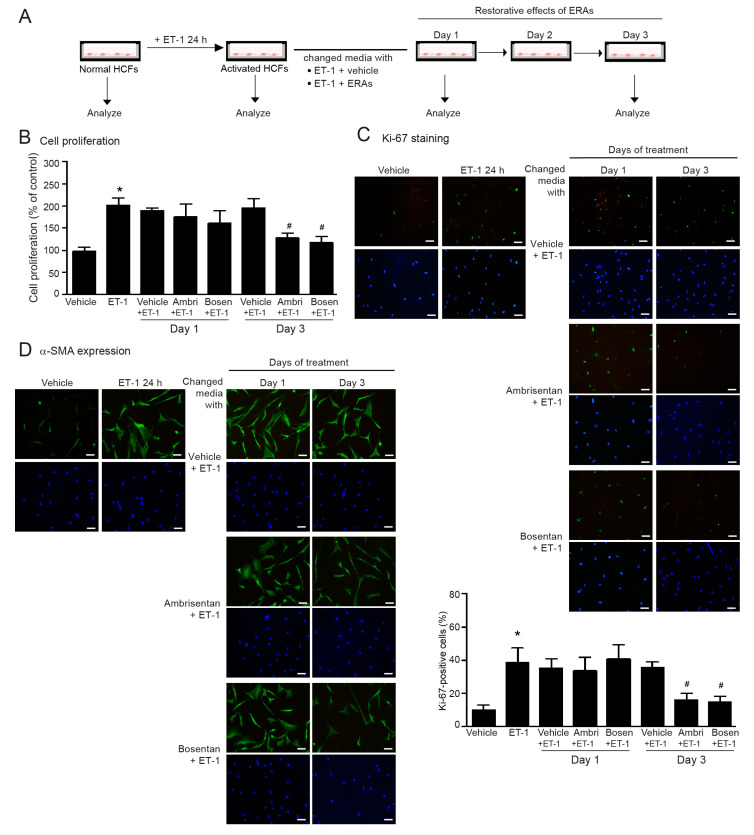
Restorative effects of ERAs on ET-1-induced cell proliferation and α-SMA synthesis. (**A**) Schematic diagram representing timeline of cell culture and treatment. (**B**–**D**) Cells were treated with 20 nM ET-1 for 24 to induce myofibroblast differentiation. After treatment for 24 h, the media were changed to either ET-1 plus vehicle, ET-1 plus ambrisentan, or ET-1 plus bosentan. Cells were further cultured for 3 days. The analysis of cell proliferation and α-SMA expression was taken on day 1 or 3 after co-treatment. (**B**) Cell proliferation was calculated as the percentage relative to the control group. (**C**) Cells were stained for Ki-67 (green) and nucleus DAPI (blue). Scale bar, 10 µm. Proliferative capacity was calculated as the percentage of Ki-67-positive cells. (**D**) α-SMA protein expression was visualized by fluorescent microscope. Cells were stained for α-SMA (green) and nucleus DAPI (blue). Scale bar, 10 µm. Data were expressed as the mean ± SD. (*n* = 4). * p < 0.05 vs. vehicle; ^#^ *p* < 0.05 vs. ET-1.

It has been shown that anti-fibrotic agents such as prostaglandin E_2_ (PGE_2_) [34] and metformin [35] can reverse the myofibroblast phenotype of lung fibroblasts. However, the effects of ERAs on the reversibility of myofibroblasts of human cardiac fibroblasts are unknown. We investigated the restorative effects of ambrisentan and bosentan on ET-1-mediated fibroblast proliferation and α-SMA synthesis. Firstly, normal fibroblasts were activated and differentiated into myofibroblasts which were characterized by increasing proliferative capacity and overexpression of α-SMA by treatment with ET-1 for 24 h. After ET-1 stimulation, the culture medium was removed and changed with fresh medium containing ET-1 plus vehicle or ET-1 plus ERAs, and cells were further cultured for 3 days and assessed for cell proliferation and α-SMA expression at days 1 and 3 (Figure 6A).

ET-1 treatment for 24 h induced fibroblast proliferation and α-SMA expression, indicating the state of myofibroblast differentiation in human cardiac fibroblasts. After changing the medium the cells were further cultured for 1 or 3 days. In absence of ERAs, proliferative capacity and overexpression of α-SMA were persistent and stable for up to 3 days (Figure 6B–D). In contrast, co-treatment with ERAs exhibited the restorative effects in a time-dependent decrease in proliferative capacity and α-SMA expression. The maximal restorative effects of ambrisentan and bosentan were observed at day 3 after ERAs addition (Figure 6B–D). These results suggested that blockade of ET_A_Rs with ERAs exhibits restorative effects on the reversal of myofibroblast phenotypes, as shown by a reduction in the proliferative capacity and α-SMA expression.

## 3. Discussion

Endothelin comprises three isoforms, ET-1, ET-2, and ET-3; ET-1 is mainly synthesized by endothelial cells and in cardiomyocytes as well as cardiac fibroblasts [36]. ET-1 is a potent vasopressor and exerts a positive inotropic effect on the human heart [37]. However, prolonged and overstimulation of ETRs with ET-1 plays a critical role in heart abnormalities, including cardiac fibrosis. In the heart of rats with myocardial infarction, myofibroblasts found at the site of infarction expressed preproendothelin-1, endothelin-converting enzymes and ETRs [38]. The synthesized ET-1 induced collagen I production [38]. These data indicated that the ET-1 system plays a critical role in cardiac fibrosis and remodeling, therefore, blockade of ETR signaling by using specific inhibitors is of intense interest because they represent the potential treatment and prevention of fibrosis in the heart.

ET-1 is able to induce myofibroblast phenotypes from resident fibroblasts, which are characterized by α-SMA and collagen overexpression [22]. In addition, the myofibroblast phenotype exhibits several capabilities, such as contractility, migration, proliferation, and production and deposition of ECM proteins such as collagens [22]. For instance, ET-1 induced α-SMA and collagen synthesis through the ET_A_Rs, but not ET_B_Rs in lung fibroblasts [16]. Treatment with ET-1 induced cell proliferation and α-SMA synthesis in alveolar fibroblasts, and neutralization of ET-1 with a monoclonal antibody was able to inhibit fibroblast proliferation [39]. Elevation of ET-1 levels was found in bronchoalveolar lavage fluid (BALF) from patients with systemic sclerosis, and this BALF containing ET-1 induced an increase in the proliferative capacity of lung fibroblasts [18]. Consistent with these previous studies, in this report we demonstrated that prolonged treatment with ET-1 up to 48 h increased proliferative capacity and induced myofibroblast differentiation as determined by α-SMA and collagen I overexpression in human cardiac fibroblasts. Even though α-SMA and collagen I are widely used for myofibroblast identifications, the other biomarkers, such as actin stress fiber formation, should be used to confirm the myofibroblast phenotype.

There are two subtypes of ETR, ET_A_R and ET_B_R, which are found in cardiac fibroblasts [40]. Both are members of the GPCR superfamily, which transduces the signals mediated through heterotrimeric G proteins affecting cardiac functions and cardiovascular disorders, including hypertension, myocardial hypertrophy, and fibrosis [22]. ET-1 induced DNA synthesis through the ET_A_R subtype in rat cardiac fibroblasts, contributing to fibroblast proliferation [33]. In addition, ET-1 induced upregulation of CTGF synthesis via ET_A_Rs in VSMCs [24]. Blockade of ET_A_R with BQ123 (a specific ET_A_R antagonist) inhibited ET-1-induced protein synthesis in cardiac myocytes, while BQ788 (a specific ET_B_R antagonist) had no effect [41]. In neonatal rat cardiomyocytes, treatment with ET-1 induced myocyte hypertrophy and upregulation of hypertrophic genes in an ET_A_R-dependent way [25]. Although most of the detrimental effects of ET-1 are mediated via the ET_A_R, the ET_B_R may be also important. Both ET_A_Rs and ET_B_Rs induced vasoconstriction of human resistance and capacitance vessels [42]. The actions of endothelin on the upregulation of collagens were mediated through ET_A_Rs and ET_B_Rs, while their actions of the downregulation of collagenase seem to be mediated through ET_A_Rs [43]. Even though the ET_B_Rs are also required for vasoconstriction and collagen synthesis, and ET_B_Rs were upregulated in fibrotic-related diseases, such as scleroderma-associated fibrotic lung disease [44] and pulmonary arterial hypertension [45], our present study revealed that the ET_B_Rs did not involve in the profibrotic effects of ET-1 in human cardiac fibroblasts.

ET-1 binding to the ETRs leads the dissociation of activated G_αq_ protein from the G_βγ_ dimer, resulting in an activation of PLC and subsequent generation of IP_3_ and DAG [19]. Elevation of IP_3_ levels leads to the activation of the Ca^2+^-dependent signaling pathway, while DAG induces translocation of PKC, resulting in Ras-mediated ERK1/2 activation in cardiac myocytes [20,46]. Furthermore, ERK1/2 activation by ET-1 was abolished by YM-254890 (a G_αq/11_ protein inhibitor), indicating the important role of the G_αq/11_ protein for ET-1-induced ERK1/2 cascade in rat L6 myoblasts [21]. Not only the G_αq_ protein, but also ETRs coupled with other isoforms, such as G_αi_ proteins, indicating that ETRs might transduce the distinct signaling pathway via multiple types of heterotrimeric G proteins [19,22]. In addition to the G_α_ protein-dependent pathway, the G_βγ_ dimer can transduce the signaling by itself. For example, the G_βγ_ subunit of ET_B_R can provoke the PI3K/Akt/eNOS signaling pathway, resulting in nitric oxide production in endothelial cells [23]. Using specific inhibitors of heterotrimeric G proteins, we found that cell proliferation, and α-SMA and collagen I synthesis induced by ET-1 were abolished by FR900359 (G_q_ inhibitor), but not PTX (G_i_ inhibitor), or gallein (G_βγ_ inhibitor) (Figure 3) Our data imply that ET_A_R stimulation induced fibroblast activation and myofibroblast differentiation through a G_αq_-dependent pathway. Interestingly, the ETR/G_αq_ axis has been reported to stimulate ERK1/2 activity through the transactivation of platelet derived growth factor receptors (PDGFRs) [21] in rat myoblasts and epidermal growth factor receptors (EGFRs) in various types of cells, including cardiomyocytes [47] and rat-1 fibroblasts [48]. However, the role of ET-1 on transactivation of these growth factor receptors in human cardiac fibroblasts is not known. It will require further studies to identify the precise mechanistic details of ETR/G_q_ compartmentation and whether growth factor receptors are assembled within this complexity for ERK1/2 activation.

Among three MAPKs, ERKs have extensively involved in the fibrosis and hypertrophy. In VSMCs, inhibition of ERK1/2 activity markedly suppressed ET-1-induced CTGF synthesis, whereas blockade of p38 MAPK had no effect [24], indicating that ET-1 regulates CTGF, which is a profibrotic mediator in an ERK-dependent pathway in vascular diseases. In addition, blockade of ERK activity using U0126 inhibited ET-1-induced ET-1 gene expression, while inhibition of either JNK or p38 MAPK had no effect, indicating that the Ras-Raf-ERK signaling pathway is necessary for ET-1 action in rat cardiac fibroblasts [33]. In agreement with these studies, our study reported that inhibition of ERK1/2 activity attenuated ET-1-induced fibroblast proliferation and myofibroblast differentiation, while inhibition of JNK and p38 MAPK had no effect. Antagonism of ET_A_Rs and blockade of G_αq_-dependent signaling were able to inhibit ET-1-induced ERK1/2 phosphorylation in human cardiac fibroblasts (Figure 4E,F). Therefore, our data and other studies suggest the essential role for the ET_A_R/G_αq_/ERK pathway for ET-1-mediated cardiac fibrosis.

Although ERAs (e.g., ambrisentan, bosentan, and macitentan) are only approved for the treatment of pulmonary arterial hypertension, they have been extensively studied in both animal and clinical experiments for their essential role in the treatment of fibrotic-related fibrosis, including cardiac fibrosis. For example, treatment with ERAs reduced cardiac remodeling in animal models of infarctive and hypertensive cardiac fibrosis [27,28]. Bosentan improved cardiac functions and reduced infarct size after myocardial ischemia/reperfusion injury in rats [28]. Furthermore, administration of bosentan improved cardiac functions, leading to hemodynamic benefits in patients with HF [30]. Here we also demonstrated that ambrisentan and bosentan suppressed ET-1-induced fibroblast activation and myofibroblast differentiation in human cardiac fibroblasts. Both ERAs showed similar preventive effects, and could help to prevent cardiac fibrosis induced by ET-1.

Unfortunately, many patients with fibrotic disorders, including HF, expressed signs and symptoms and reached clinical care after significant fibrosis has already advanced [1,49]. Interestingly, many studies have reported that the myofibroblast differentiation process can be reversed [50,51]. For these HF patients, reversal of myofibroblast differentiation (so called dedifferentiation) represents attractive therapeutic strategies to already established myocardial fibrosis [52]. Even though many anti-fibrotic agents possess the ability to reverse the myofibroblast differentiation in lung fibroblasts such as PGE_2_ [34] and metformin [35], the effects of ambrisentan and bosentan on the reversal of myofibroblast differentiation are unknown. In our present study, we report that ambrisentan and bosentan promote dedifferentiation of myofibroblasts after day 3 of treatment, with loss of proliferative capacity, and reduce the expression of α-SMA, indicating the restorative effects of these ERAs in human cardiac fibroblasts (Figure 6). These preventive and restorative effects of ERAs provide the current trends of approach in attenuating and recovering cardiac fibrosis and could potentially be achieved. It should be noted that our results have come from the study using cardiac fibroblasts. The animal models of myocardial fibrosis should be performed to confirm the underlying mechanism of profibrotic effects of ET-1 as well as the preventive and restorative effects of ERAs.

## 4. Materials and Methods

### 4.1. Materials

Ambrisentan, bosentan, pertussis toxin (PTX), 3-(4,5-dimethylthiazol-2yl)-2,5-diphenyl-2H-tetrazolium bromide (MTT), SB203580 (p38 MAPK inhibitor), SP600125 (JNK inhibitor), and FR180204 (ERK1/2 inhibitor) were obtained from Sigma-Aldrich (Saint Louis, MO, USA). ET-1, BQ788 (ET_B_R antagonist), and gallein (G_βγ_ inhibitor) were purchased from Tocris Bioscience (Ellisville, MO, USA). FR900359 (G_αq_ inhibitor) was obtained from Cayman Chemical (Ann Arbor, MI, USA). Fibroblast medium and related cell culture reagents were purchased from Cell Applications (San Diego, CA, USA). Ambrisentan, bosentan, SB2033580, SP600125, FR180204, gallein, and BQ788 were dissolved in dimethyl sulfoxide (DMSO), whereas endothelin-1 and PTX were dissolved in distilled water when preparing the stock solutions. Aliquots of stock solution were stored at −20 °C.

### 4.2. Cell Culture

Human cardiac fibroblasts (catalog no. 306-05f) were obtained from Cell Applications and were cultured at 37 °C/5% CO_2_ in fibroblast growth medium containing 1% penicillin/streptomycin (P/S) solution as described previously [53]. The cells between passages 3 and 5 were subjected to all experiments.

### 4.3. Measurement of Cell Proliferation by MTT Assay

Fibroblasts (5 × 10^3^ cells/well) were cultured in 96-well plates containing medium supplemented with 1% growth serum and 1% P/S. Afterward, fibroblasts were incubated with specific inhibitors for 1 h before stimulation with ET-1 for 24 or 48 h. After stimulation, the medium was discarded and cells were further incubated with MTT solution (1 mg/mL) for 4 h. After incubation, dimethyl sulfoxide (100 μL/well) was added and thoroughly mixed to dissolve the formazan crystals [11]. Finally, the absorbance of the solution was monitored by an EZ Read 400 microplate reader (Biochrom, Cambridge, UK) at 570 nm wavelength. The formula below was used to calculate the numbers of viable cells.

Cell viability (%) = (Absorbance of treated cells/Absorbance of non-treated cells) × 100.

### 4.4. Measurement of Proliferative Capacity by Ki-67 Staining Assay

Ki-67 is present in all active phases of cell cycle (e.g., G1, S and G2), and mitosis, but is absent in G0 phase (resting state). Ki-67 is used for determining the proliferative capacity of the cells. Fibroblasts (1 × 10^4^ cells/well) were cultured in 12-well plates containing gelatin-coated coverslips. After stimulation, cells were fixed with 4% paraformaldehyde (PFA) overnight and washed with phosphate-buffered saline (PBS) before permeabilized with 0.1% Triton X-100 solution for 5 min. Cells were washed with PBS, incubated with 1% bovine serum albumin (BSA) for 30 min, and stained with Ki-67 conjugated-FITC antibody (1:200, Invitrogen) for 1 h. After washing, the coverslips containing cells were mounted onto microscope slides with DAPI containing Antifade Mountant (Invitrogen, Carlsbad, CA, USA). The samples were imaged by fluorescence microscope (Nikon Eclipse Ts2R; 20X objective lens, Tokyo, Japan) at 488/520 nm for excitation and emission wavelength, respectively. Three to five different images were taken per each group of treatment. Cell proliferation indices were determined by counting at least 100 cells from 3–4 randomly selected images. The Ki-67 score is defined as the ratio of Ki-67-positive cells (green) to the total counted cells.

### 4.5. Detection of α-SMA Expression by Fluorescence Microscopy

Fibroblasts (1 × 10^4^ cells/well) were cultured in 12-well plates containing gelatin-coated cover slips. After stimulation, cells were fixed with 4% PFA overnight and washed with PBS before permeabilized with 0.1% Triton X-100 for 5 min [54]. After blocking with 1% BSA for 30 min, cells were incubated with anti-α-SMA antibody (1:250, Sigma-Aldrich) for 1 h. After washing, cells were incubated with anti-mouse Alexa Fluor 488 secondary antibody (Invitrogen) for 1 h. After washing, the coverslips containing cells were mounted onto microscope slides with DAPI containing Antifade Mountant (Invitrogen). The α-SMA protein expression was detected by fluorescence microscope (Nikon Eclipse Ts2R; 20X objective lens) at 488/520 nm. Three to five different images were taken per each group of treatment.

### 4.6. Western Blotting

Fibroblasts (2 × 10^5^ cells/well) were cultured in 6-well plate. Cells were pretreated with various inhibitors before stimulation with ET-1 in serum-free media condition. After stimulation, cells were rinsed with cold PBS, then lysed with Triton X-100 lysis buffer (containing: 0.8% Triton X-100, 20 mM Tris-HCl, 2 mM EDTA, 150 mM NaCl, and protease inhibitor cocktail) for 2 h with gentle rotation, followed by centrifugation at 14,000 rpm at 4 °C for 15 min [55]. The supernatants were then collected, and protein levels were measured by Bradford protein assay (Bio-Rad, Hercules, CA, USA). The equal amounts of protein samples in SDS loading buffer were heated for 5 min, and subjected to 10% SDS-PAGE. After that, protein samples from gels were transferred to PVDF membranes and incubated with 2% BSA for 1 h. After blocking with 2% BSA, membranes were incubated with primary antibodies against p-ERK-1/2 (1:2000, Cell signaling, Danvers, MA, USA), ERK-1/2 (1:2000, Cell signaling), and β-actin (1:3000, Cell signaling) overnight. After washing with tris-buffered saline with Tween 20 (TBST), membranes were incubated with anti-rabbit HRP-conjugated antibody (1:3000, Amersham Biosciences, Buckinghamshire, UK). After washing with TBST, membranes were soaked in chemiluminescence substrate (SuperSignal West Pico PLUS, Thermo Scientific, Waltham, MA, USA) and then exposed to a GelDoc XR imaging system (Bio-Rad). The band intensity was analyzed using Image J software (version 1.53k, NIH, Bethesda, MD, USA).

### 4.7. mRNA Expression Analysis by Real-Time qRT-PCR

Total RNA was extracted with GeneJET RNA isolation kits (Thermo scientific). The qRT-PCR was performed by the AriaMx Real Time PCR system (Agilent Technologies, Santa Clara, CA, USA) using the Brilliant III ultra-fast SYBR green qRT-PCR master mix (Agilent Technologies) [56]. The human primers were designed and purchased from Macrogen (Seoul, Republic of Korea) as follow; α-SMA (sense, 5′- tggctattccttcgttactactgct-3′; antisense, 5′-catcaggcaactcgtaactcttctc-3′), collagen I (sense, 5′-ctgctggacgtcctggtgaa-3′; antisense, 5′-acgctgtccagcaataccttgag-3′), and β-actin (sense, 5′-gtggccgaggactttgattg-3′; antisense, 5′-agtggggtggcttttaggatg-3′). The α-SMA and collagen I mRNA levels were normalized to β-actin and expressed as the fold over the vehicle-treated group based on the comparative cycle threshold (2^−ΔΔCT^) assay [57].

### 4.8. Data Analysis

The results were expressed as the mean ± SD from 4 independent experiments. Statistical significance among different groups was determined by Student’s t-test and one-way ANOVA with Tukey’s post hoc test. The statistical analyses were evaluated with GraphPad Prism^TM^ (version 6.0). A *p* value less than 0.05 (*p* < 0.05) was considered to represent significant differences.

## 5. Conclusions

**Figure 7:**
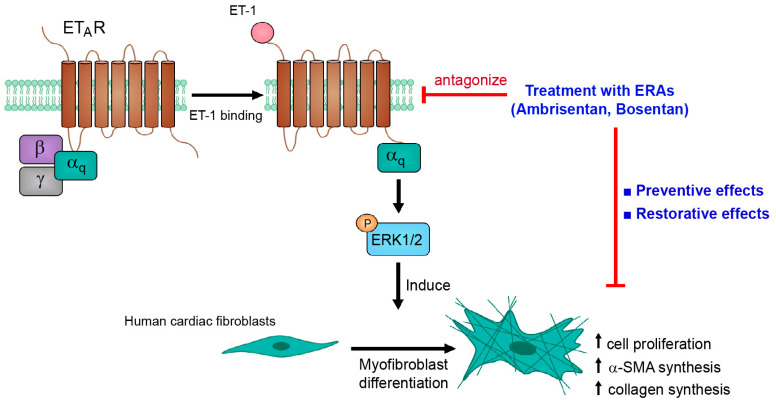
Schematic representing the ET_A_R/G_αq_/ERK signaling pathway for ET-1-inducd fibroblast activation and myofibroblast differentiation. ET-1 binding to ET_A_Rs leads to an activation of heterotrimeric G_q_ protein, resulting in the dissociation of the G_αq_ protein from the G_βγ_ subunit. The activated G_αq_ proteins transduced the signal through ERK1/2, which in turn induced fibroblast activation and myofibroblast differentiation. Interestingly, ETR antagonism using ERAs, ambrisentan and bosentan, exhibits cardioprotective effects by prevention and restoration of fibrotic actions of ET-1 in human cardiac fibroblasts. α-SMA: α-smooth muscle actin; ET-1: Endothelin-1; ERAs: endothelin receptor antagonists.

The results of our present study provide an essential role for the ERK1/2 signaling pathway for ET-1-induced fibroblast activation and myofibroblast differentiation in human cardiac fibroblasts. The ET-1 effects appear to be predominantly mediated by the ET_A_R subtype. Moreover, G_αq_ protein is required for ET_A_R-mediated ERK1/2 activation (Figure 7). Our data support the concept whereby ET_A_R stimulation contributes to the progression of cardiac fibrosis in HF patients. In addition, ETR antagonism using ERAs, ambrisentan and bosentan, exhibits cardioprotective effects by prevention and restoration of ET-1-induced fibroblast proliferation and α-SMA synthesis.

## Data Availability

All data generated or analyzed during the current study are included in this published article and its supplementary information file.

## Supplementary Materials

The following supporting information can be downloaded at: https://www.mdpi.com/article/10.3390/ijms24054475/s1.
