# Supplementary material for: Endothelin-1 Induces Cell Proliferation and Myofibroblast Differentiation through the ETAR/Gαq/ERK Signaling Pathway in Human Cardiac Fibroblasts
Source: Int J Mol Sci. 2023 Feb 24;24(5):4475. doi: 10.3390/ijms24054475 (PMC10002923; doi:10.3390/ijms24054475)

Supplementary information

Supplementary figures

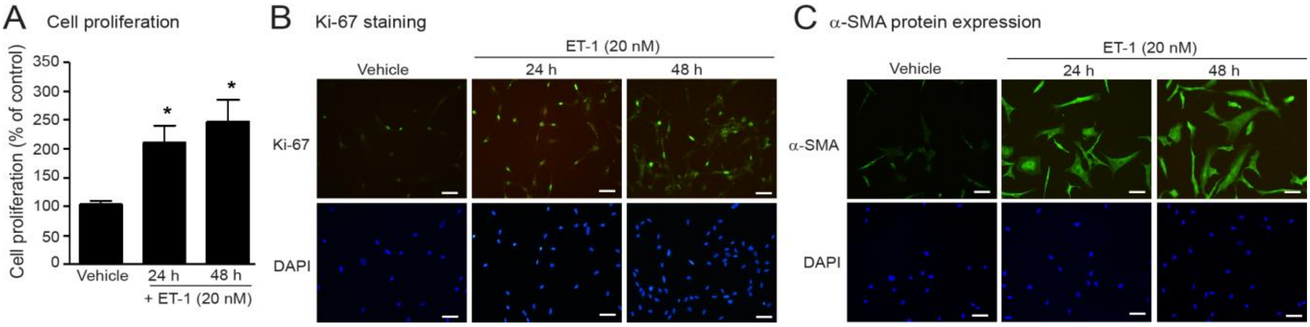

Figure S1. Treatment with ET-1 up to 48 h induced cell proliferation and α-SMA expression
(A-B) Cells were treated with 20 nM ET-1 for the indicated time. (A) Cell proliferation was expressed as the percentage relative to vehicle-treated group, and shown as the mean ± SD. **P*<0.05 versus vehicle. (B) Proliferative capacity of fibroblasts was determined by Ki-67 immunofluorescence assay. Cells were stained for Ki-67 (green) and nucleus DAPI (blue). Scale bar represents 10 μm. (C) α-SMA protein expression was visualized by fluorescent microscope. Cells were stained for α-SMA (green) and nucleus DAPI (blue). Scale bar represents 10 μm. Data are obtained from 3 independent repetitions (N = 3).

Cell cytotoxicity of specific inhibitors

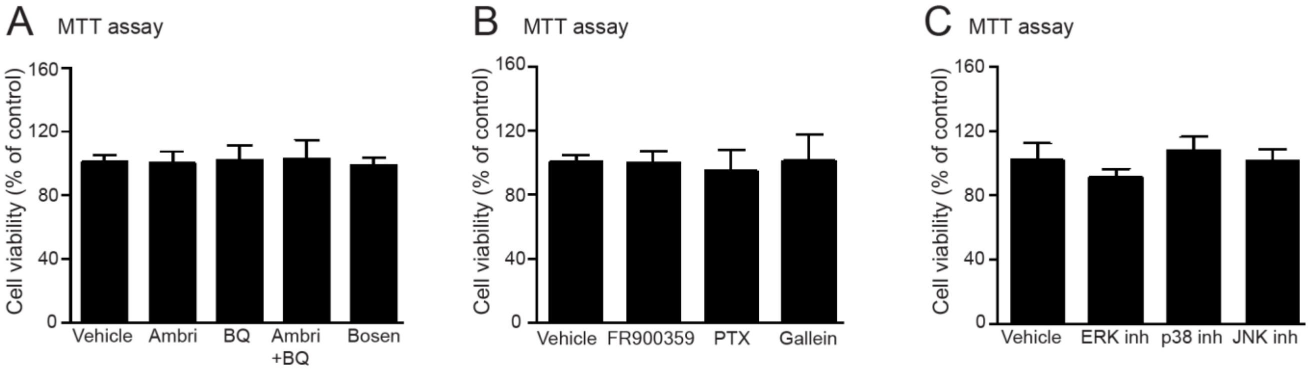

Figure S2. Effects of each inhibitor on cell cytotoxicity as determined by MTT assay
(A-C) Cells were treated with each inhibitor at the indicated concentrations for 48 hours. Cell viability was quantified and expressed as the percentage of cell viability. Data are shown as the mean ± SD from 3 independent repetitions (N = 3).

Figure S3. The whole uncropped images of western blots presented in Figure 4E and 4F

The whole blott at specific molecular weight for p-ERK-1/2 (44, 42 kDa), ERK-1/2 (44, 42 kDa), and β-actin (45 kDa)

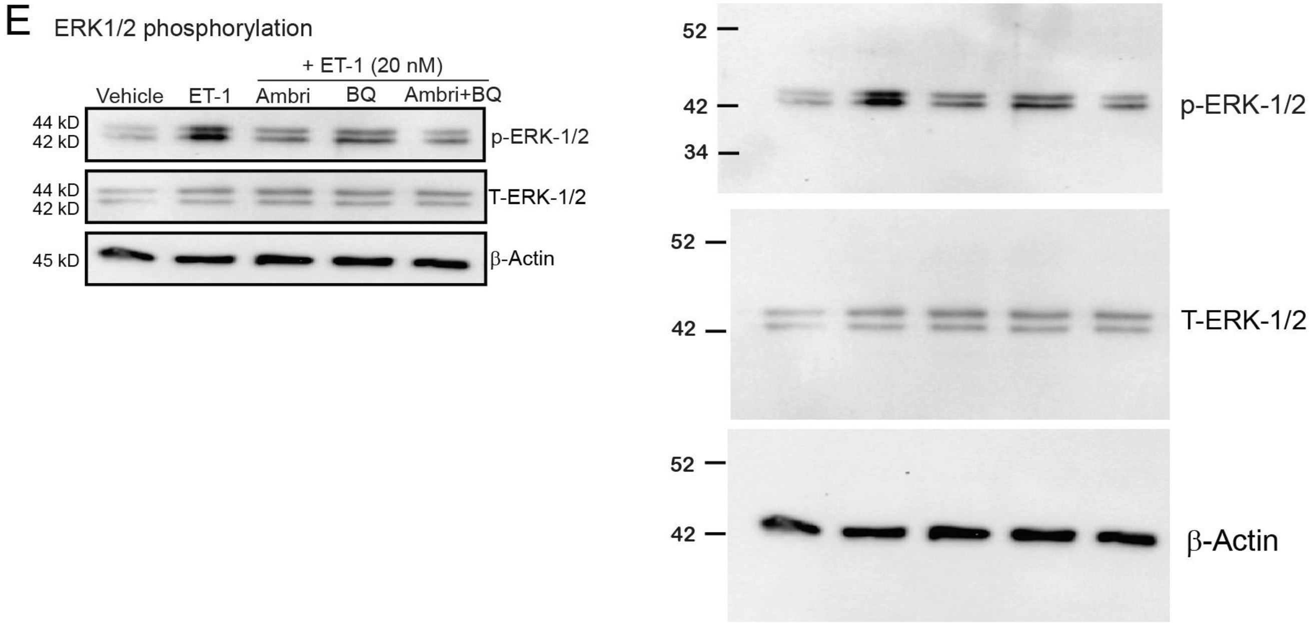

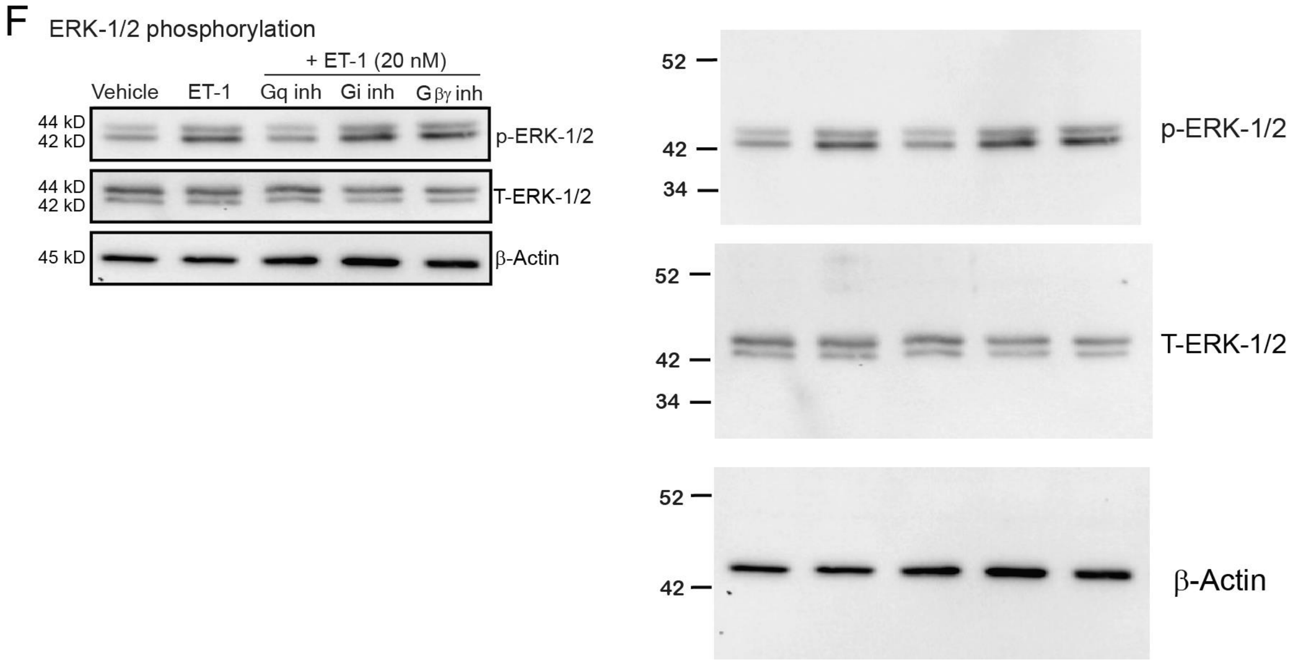

Figure 4E

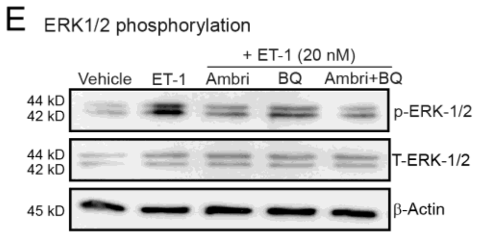

The whole blott at specific molecular weight for p-ERK-1/2 (44, 42 kDa), ERK-1/2 (44, 42 kDa), and β-actin (45 kDa)

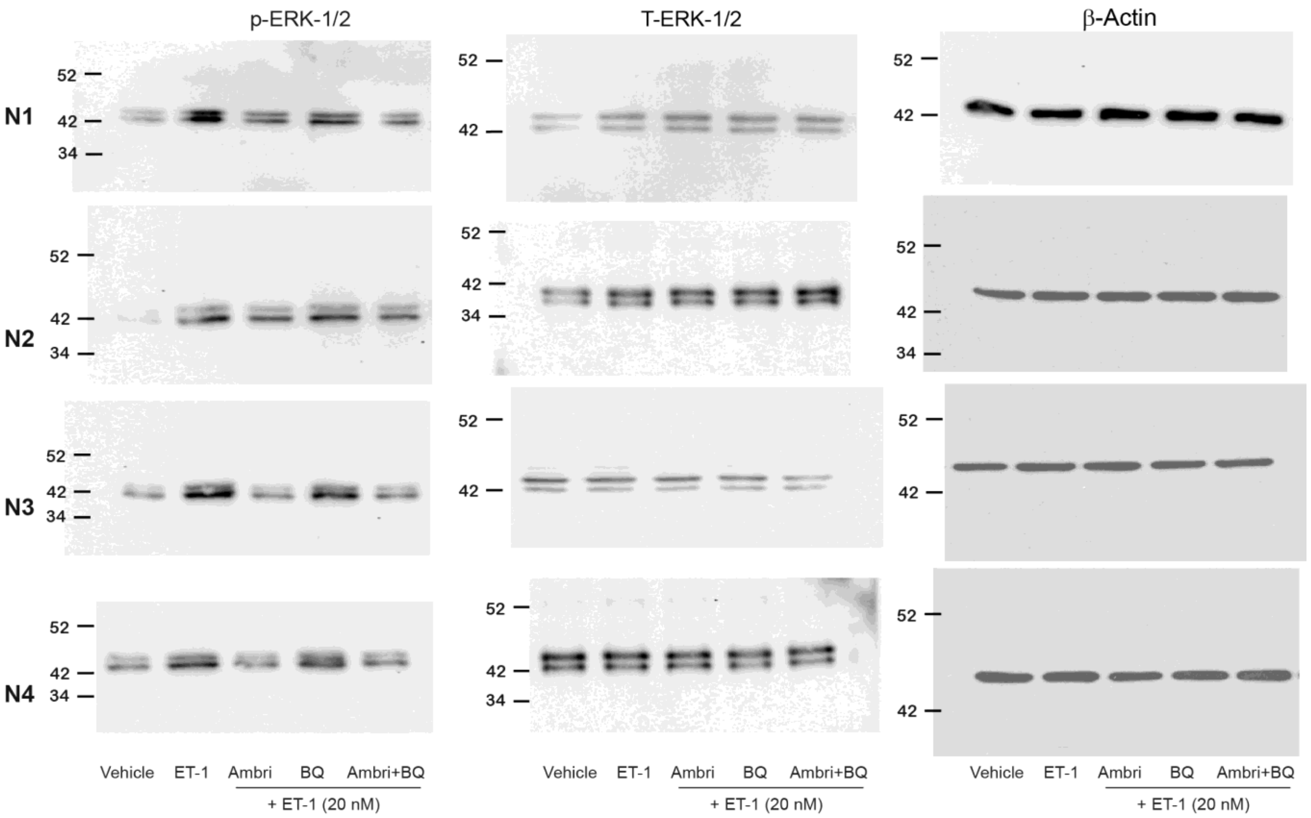

Figure 4F

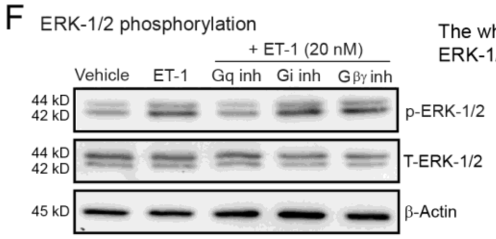

The whole blott at specific molecular weight for p-ERK-1/2 (44, 42 kDa), ERK-1/2 (44, 42 kDa), and β-actin (45 kDa)

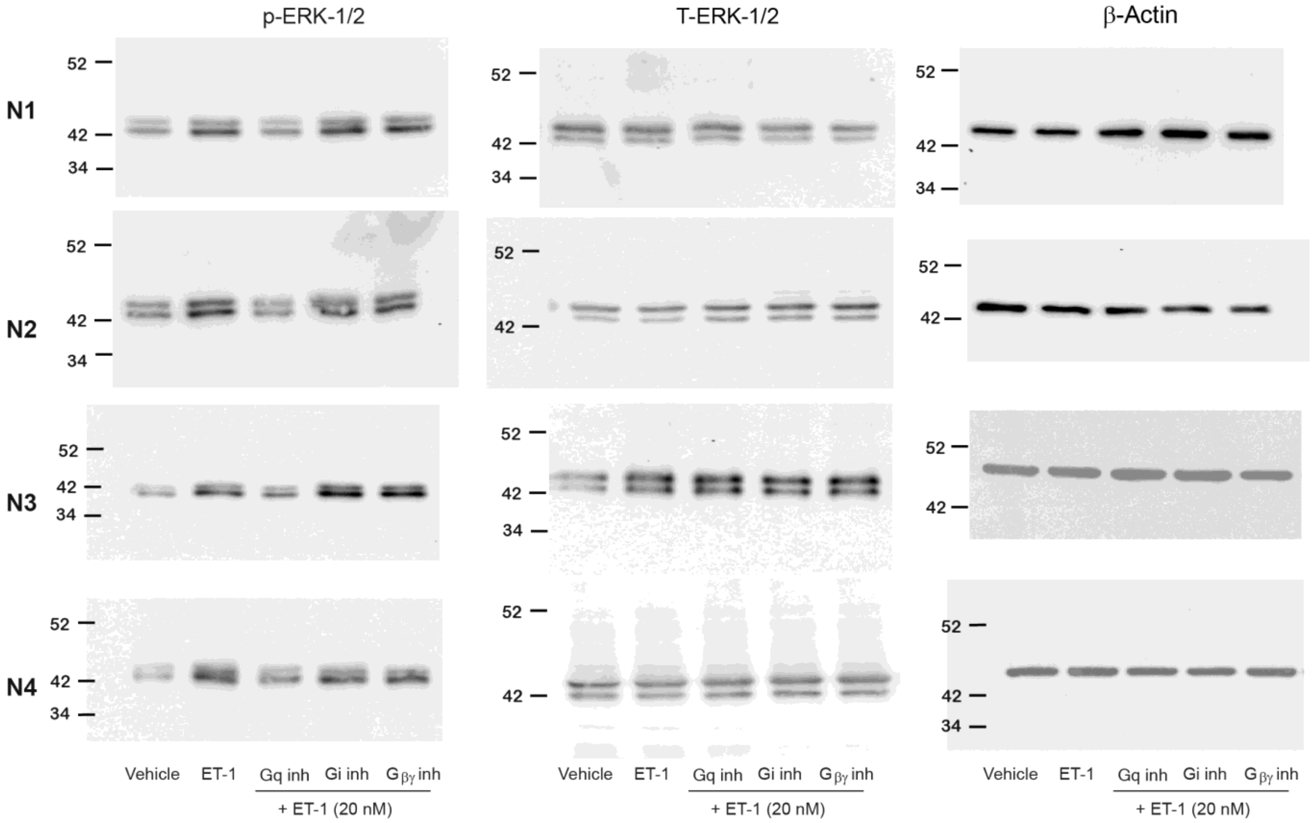

Supplement: Supplementary file 1 [file ijms-24-04475-s001.zip › ijms-2193931-supplementary.pdf]
